# Are Human Peripheral Nerves Sensitive to X-Ray Imaging?

**DOI:** 10.1371/journal.pone.0116831

**Published:** 2015-03-10

**Authors:** Jonas Francisco Scopel, Luciano de Souza Queiroz, Francis Pierce O’Dowd, Marcondes Cavalcante França Júnior, Anamarli Nucci, Marcelo Gonçalves Hönnicke

**Affiliations:** 1 Instituto de Ciências da Saúde, Universidade Federal de Goiás, Jataí, Goiás, 75804-020, Brazil; 2 Departamento de Anatomia Patológica, Universidade Estadual de Campinas, Campinas, São Paulo, 13083-887, Brazil; 3 Laboratório Nacional de Luz Síncrotron, Campinas, São Paulo, 13083-970, Brazil; 4 Departamento de Neurologia, Universidade Estadual de Campinas, Campinas, São Paulo, 13083-887, Brazil; 5 Instituto Latino-Americano de Ciências da Vida e da Natureza, Universidade Federal da Integração Latino-Americana, Foz do Iguaçu, Paraná, 85867-970, Brazil

## Abstract

Diagnostic imaging techniques play an important role in assessing the exact location, cause, and extent of a nerve lesion, thus allowing clinicians to diagnose and manage more effectively a variety of pathological conditions, such as entrapment syndromes, traumatic injuries, and space-occupying lesions. Ultrasound and nuclear magnetic resonance imaging are becoming useful methods for this purpose, but they still lack spatial resolution. In this regard, recent phase contrast x-ray imaging experiments of peripheral nerve allowed the visualization of each nerve fiber surrounded by its myelin sheath as clearly as optical microscopy. In the present study, we attempted to produce high-resolution x-ray phase contrast images of a human sciatic nerve by using synchrotron radiation propagation-based imaging. The images showed high contrast and high spatial resolution, allowing clear identification of each fascicle structure and surrounding connective tissue. The outstanding result is the detection of such structures by phase contrast x-ray tomography of a thick human sciatic nerve section. This may further enable the identification of diverse pathological patterns, such as Wallerian degeneration, hypertrophic neuropathy, inflammatory infiltration, leprosy neuropathy and amyloid deposits. To the best of our knowledge, this is the first successful phase contrast x-ray imaging experiment of a human peripheral nerve sample. Our long-term goal is to develop peripheral nerve imaging methods that could supersede biopsy procedures.

## Introduction

The estimated peripheral neuropathy prevalence in the general population is approximately 2%, but it can achieve 8% in adults over 55 years of age [1]. Because neuropathies have several causes, assessment and diagnosis may become challenging. Thus, even with a proper assessment, that includes nerve conduction studies, between 25% and 40% of all neuropathies are still labelled idiopathic [2,3]. Furthermore, functional studies have limitations to assess the exact location, cause and extent of nerve damage and concomitant disease of surrounding tissues. In these cases, imaging can provide an accurate morphologic correlation to functional data, and make patient care more effective in a variety of pathological conditions, such as entrapment syndromes, traumatic injuries, and space-occupying lesions [4]. Ultrasound (US) and magnetic resonance imaging (MRI) imaging are becoming useful methods for this purpose [5], but their main disadvantage is a limited spatial resolution.

In this scenario, the advent of new x-ray imaging methods, joined with the development of high spatial resolution x-ray imaging detectors, could greatly improve image quality and reduce radiation exposure over conventional techniques. The method, termed phase contrast x-ray imaging (PCI), explores the real part of the refraction index, besides the imaginary part (responsible for the photoelectric absorption), to enhance the contrast. The real part of the refraction index can be explored by the deformation of the x-ray wavefront when passing through the object (propagation-based x-ray phase contrast imaging) [6–9], or by the deviations of the beam when passing by the sample, which can be explored by phase (x-ray interferometer, Talbot-Lau interferometer, coded-aperture) [10–12] or by the angular deviations of the beam (diffraction enhanced imaging or analyzer-based imaging) [13–16]. PCI medical applications have received increasing interest over the past few years and efforts to implement the technique as a clinical equipment [17–21], including breast cancer investigation and joint imaging [22]. Recent studies suggest that these techniques could also be applied to nerve tissue evaluation [23–26], but only few experiments were performed so far.

In the present study, we performed synchrotron radiation (SR) propagation-based imaging (PBI) to analyze a human sciatic nerve sample. The images displayed high contrast and high spatial resolution, allowing clear identification of each fascicle structure and surrounding connective tissue. Our long-term goal is to analyze the role of PCI in the diagnosis of peripheral neuropathies, together with the construction of a knowledge base for image interpretation, so that details visualized in the phase-contrast images can be easily assigned to known lesions.

## Materials and Methods

**Fig 1:**
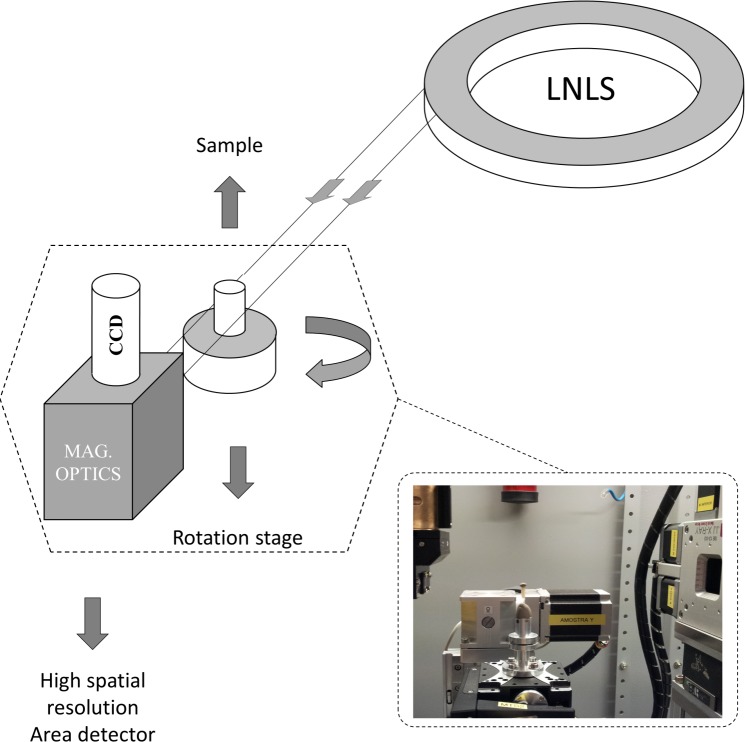
Schematic representation of experimental setup mounted into IMX beamline hutch at LNLS. In the inset it is shown a picture of the setup taken into the beamline hutch.

The experiments were carried out at a new dedicated x-ray imaging beamline (IMX) in Laboratorio Nacional de Luz Síncrotron (LNLS) (see Fig. 1). Other phase contrast X-ray imaging experiments [27,28], including diffraction enhanced imaging [29–32] and PBI [9,30] had been already carried out at LNLS, however, in a diffraction beamline which does not have well prepared optical elements specially for imaging, like IMX has. The IMX beamline is a 20 m long beamline. Presently, it works with a white x-ray beam with a spectrum between 8 and 24 keV, limited by the use of filters. In the near future, the plan is to have also an option to work with a double bounce multilayer monochromator. The x-ray source size is around 100 μm vertically (V) x 400 μm horizontally (H). The imaging detector system consists of a 50 μm thick YAG:Ce scintillator and a charged coupled device camera (PCO2000) of 2048 x 2048 pixels. A set of lenses (Optique Peter microscope) magnifies the image displayed on the scintillator before it is acquired by the charged coupled device. Different magnifications can be applied depending on the required spatial resolution. For our measurements, the images were acquired with 4× and 10× magnification optics. These magnifications give us virtual pixel sizes of 1.9 μm x 1.9 μm and 0.74 x 0.74 μm, with a field of view of 3.79 mm x 3.79 mm and 1.52 mm x 1.52 mm, respectively. Due to the modulation transfer function of the detector system, the spatial resolution of the two different magnification optics are 2.1 μm x 2.1 μm and 0.84 μm x 0.84 μm, for 4× and 10× magnification respectively.

The vertical x-ray source size coupled with the average distance from the source and, mainly, a high spatial resolution imaging detector system enable us to acquire propagation-based x-ray phase contrast images. Therefore, due to the low transverse coherence length, the images can be acquired in the edge regime.

The human sciatic nerve sample was obtained previously and stored at the University Hospital of the Universidade Estadual de Campinas (HC-UNICAMP). A written consent was obtained from HC-UNICAMP tissue bank, stating that the institution agreed in providing it for the experiments. Informed consent from patients was not demanded, since the data were analyzed anonymously and the sample was collected and stored previously. This study was submitted to Ethics Committees of UNICAMP and Universidade Federal de Goiás, and the measurements began only after its final approval (CAAE number 20055313.0.3001.5404, approval no. 603.217-0).

**Fig 2:**
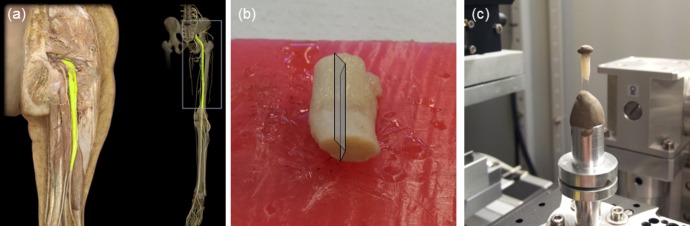
Sample preparation for the tomographic study. (a) anatomical position of human sciatic nerve; (b) wedge-shaped cut of the sciatic nerve, allowing it to fit the detector field of view; (c) approximately 3 mm thick sample [preserved in formalin solution (10%)] inside the boron silicate capillary tube, positioned for tomographic image acquisition.

The sciatic nerve sample was preserved in a formalin solution (10%) and had a cylindrical shape (diameter ∼ 1 cm and length ∼ 3 cm). It was imaged by propagation-based x-ray phase contrast radiography and tomography, in edge-detection regime [7]. For radiographs, a portion of the nerve was embedded in paraffin, and 10 μm thick longitudinal and axial sections were cut. To avoid huge x-ray attenuation by the use of conventional glass microscope slides, the sample was mounted on polyurethane slides. For tomographic reconstruction, another portion of the nerve, still preserved in formalin solution (10%) and not embedded in paraffin, was cut into wedge-shaped to fit the detector field of view, and inserted into a boron silicate capillary tube with an internal diameter of 3 mm (see Fig. 2). The capillary tube was tapped in order to avoid nerve shrinkage during the tomography scan. A high precision sample rotation stage was mounted on top of a translation stage to set the sample in and out of the beam path. The sample to detector distance was set to 70 mm. The radiography and tomography doses were not measured. However, the absorbed dose (D) was calculated following the equation given elsewhere [33], therefore, modified for a polychromatic x-ray beam, as follows:

$$D=\int_{E_{0}}^{E_{f}}\phi_{0}(E_{ph}).\left[\frac{\mu }{\rho }(E_{ph})\right].dE_{ph}$$ (1)

where E_0_ and E_f_ are the start and final x-ray spectrum energies, Φ_0_(E_ph_) is the photon flux on area A, μ/ρ(E_ph_) is the mass energy absorption coefficient and dE_ph_ is the differential photon energy.

For comparison, optical microscopy slides were prepared using adjacent sections of the nerve portions used in radiography and tomography. They were cut in 4 μm thick sections, embedded in paraffin, and stained with hematoxylin and eosin. The images were acquired by a light photomicroscope, with the same magnification of the radiographic images (10× for radiographs and 4× for tomography).

The radiographs were acquired with an exposure time of 160 ms, and a 10× magnification optics. For tomography scans, 1000 projections were taken, equiangularly sampling the range from 0° to 180°. The exposure time for each projection was 1.1 seconds, with 4× magnification optics. In order to reduce image noise, twenty adjacent computed tomography (CT) slices were summed up.

## Results

### Phase contrast radiography

**Fig 3:**
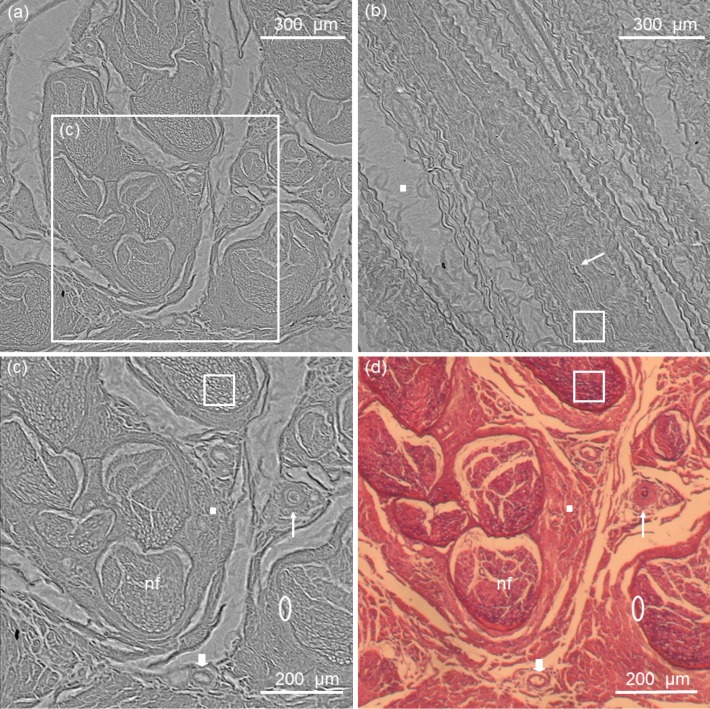
Phase contrast radiography of human sciatic nerve in axial (a) and longitudinal sections (b). Dose in each radiographic image was 0.2 μGy. Detail of the axial section is shown in (c), with the corresponding optical microscope image in (d). Phase contrast radiography shows nerve fascicles (nf), arterioles (thin arrow), venules (thick arrow), epineurium (solid square), perineurium (ellipse) and nerve fibers (hollow square). For validation of the structures, the corresponding optical microscope image is shown in (d).

Phase contrast radiography showed a clear distinction of the fascicular nerve architecture, surrounded by its connective tissue and blood vessels (see Fig. 3). Two sheets of connective tissue are easily identified: the epineurium and the perineurium. Epineurium is the outermost layer and consists of dense trabecular conjunctive tissue, involving multiple nerve fascicles and blood vessels. Perineurium is formed by lamellae of squamous cells, which constitute a barrier between axons and epineurium. A reticular pattern is visible inside each fascicle, corresponding to endoneurium and nerve fibers. Arterioles and venules, with diameter ranging from 10 to 50 μm in diameter, permeate the connective tissue, the former being distinguished by their thicker walls. Overall nerve architecture, fascicle arrangement, and even nerve fiber diameter analysis were possible to be detected through phase contrast radiography. It is worth noticing that nerve structures can only be seen in the optical microscopy images by the use of staining procedures, otherwise it would be transparent for such a technique. This is not demanded, nor usual, in phase contrast x-ray radiography.

To have a more reliable detection of the peripheral nerve structures we followed up with phase contrast x-ray tomography of an entire nerve section preserved in formalin solution (10%) as described in the next subsection.

### Phase Contrast x-ray tomography

**Fig 4:**
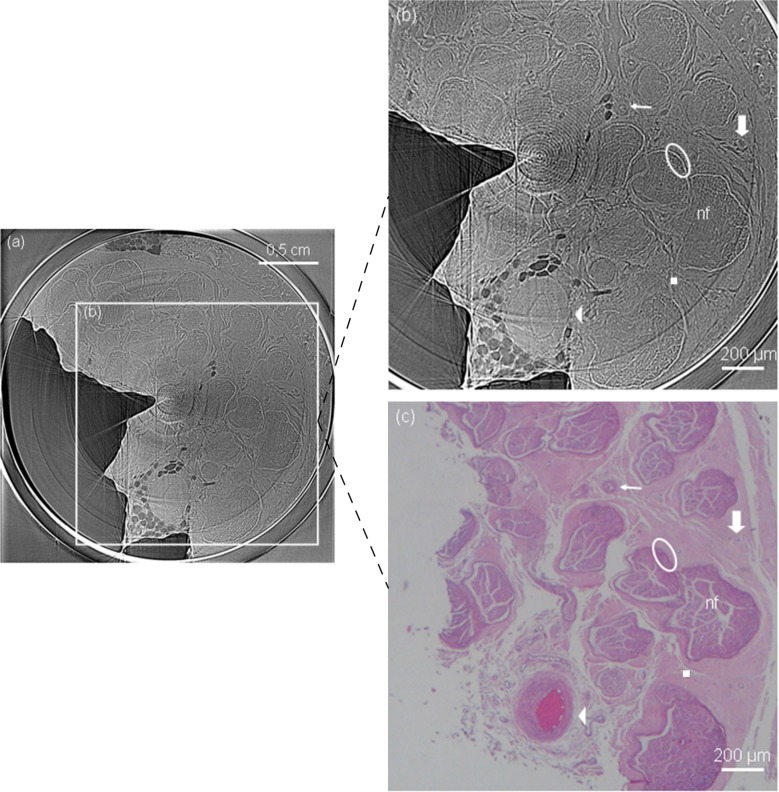
Axial slice of phase contrast x-ray tomography (a). Dose for the full tomography scan (1000 projections) was 0.23 mGy. Detailed axial section is shown in (b) and, for comparison, its corresponding optical microscope image in (c). Clear delineation of each nerve fascicle and surrounding structures are possible without sectioning, fixation or staining procedures with PC tomography. In the images, nerve fascicles (nf), an artery (arrow head), arterioles (thin arrow), venules (thick arrow), epineurium (square) and perineurium (ellipse) can be easily distinguished.

Unprecedented phase contrast x-ray tomography results allowed us to delineate fine nerve structures, with almost the same resolution and contrast of the radiography, and without the need of sectioning, fixation or staining procedures (see Fig. 4). An artery is also visible inside the nerve tissue, with its exact counterpart seen in the optical microscopy image (see Fig. 4C). Note that the elements on the optical microscopy images are slightly loosened because of paraffin wax preparation artifacts.

## Discussion

Current nerve imaging modalities include MRI and US. Although they have made important contributions to nerve imaging, such as entrapment syndromes, traumatic injuries, and space-occupying lesions, visualization of thin nerve fascicles is not always possible. High-resolution US is currently the imaging modality of choice for the examination of peripheral nerves due to its good spatial resolution [5], but may not provide enough soft tissue contrast. On the other hand, MRI imaging supplies good differentiation of soft tissue structures, but may lack spatial resolution. Novel x-ray imaging techniques could share the advantages of both methods, by joining the unrivalled x-ray imaging spatial resolution to a high soft tissue contrast [22]. Such gain could enable the detection of more subtle changes, such as Wallerian degeneration, hypertrophic neuropathy, inflammatory infiltration, leprosy neuropathy and amyloid deposits.

We presented here an attempt to image a human peripheral nerve sample. Nerve fascicles and surrounding connective sheets were clearly visible on both radiographic and tomographic images. The last show an unprecedented remark in terms of nerve imaging technique, since none of the present peripheral nerve imaging methods can resolve perineurium nor inside fascicle content so accurately.

In this initial study, we imaged a dissected nerve, without surrounding musculoskeletal tissue. To assess the eventual clinical and research potential of this technique, further study of a nerve in situ must be performed. In our trials, we experienced some minor ring artifacts on computed tomography. They were attenuated by post-processing filters, however, since the contrast of the nerve structures is not so high, they still can be seen. This could be improved by increasing soft tissue contrast, with the use other phase contrast imaging techniques, such as monochromatic PBI [7], diffraction enhanced imaging [13–20] or, even, Talbot-Lau inteferometric imaging [11,21]. Note that different portions of the same nerve were used to acquire radiographic and tomographic data. In all the radiographies as well as in the tomography slices the nerve structures were clearly visible. To assure the efficiency of this method, other samples need to be imaged. This is our goal for the following experiments on different pathological patterns.

Synchrotron-based imaging is a safe procedure. Radiation exposure can be limited with various security features. The most widely synchrotron imaging use in human subjects is coronary angiography. It has been safely performed in more than 500 patients worldwide [34]. Currently, there are limited clinical applications for SR imaging, and it remains predominantly a research tool. More recently, the technological advances in SR sources as well as new compact x-ray sources with high brightness, such as the liquid-metal-jet anode electron-impact x-ray sources [35], the plasma-based x-ray source [36], and the quasi-monochromatic tunable inverse Compton scattering x-ray sources [37], have made possible the practical implementation of PCI. These new approaches have great capacity to improve soft tissue contrast compared to conventional absorption-based x-ray imaging techniques in many clinical applications [38]. Quantitative analysis based on mathematical models indicate that it is theoretically possible through optimal design of the x-ray imaging system to achieve high spatial resolution (<100 μm) in 3D medical x-ray imaging of the human body at a clinically acceptable dose level (<10 mGy) by introducing PCI [39]. Additional evidence toward the implementation of a clinically compatible x-ray phase contrast CT system was recently demonstrated by Sarapata et al., by using a glancing-angle interferometer [40].

In summary, SR PBI represents an imaging technique that can be applied to nerve imaging, as shown by outstanding tomography results on sciatic nerve structures. Exquisite delineation of the nerve morphology was obtained in this preliminary study of PCI of a human nerve sample. To further evaluate the utility of PCI in peripheral nerve imaging, we intend to analyze diverse pathological patterns, such as Wallerian degeneration, hypertrophic neuropathy, inflammatory infiltration, leprosy neuropathy and amyloid deposits, and also perform in situ nerve imaging experiments.
